# Awareness of ovarian cancer risk factors, beliefs and attitudes towards screening: baseline survey of 21 715 women participating in the UK Collaborative Trial of Ovarian Cancer Screening

**DOI:** 10.1038/sj.bjc.6605809

**Published:** 2010-07-20

**Authors:** L Fallowfield, A Fleissig, J Barrett, U Menon, I Jacobs, J Kilkerr, V Farewell

**Affiliations:** 1 CRUK Psychosocial Oncology Group, Brighton & Sussex Medical School, University of Sussex, Falmer BN1 9QG, UK; 2 MRC Biostatistics Unit, Institute of Public Health, University Forvie Site, Robinson Way, Cambridge CB2 0SR, UK; 3 Gynaecological Oncology, University College London, EGA Institute for Women's Health, London W1T 7DN, UK

**Keywords:** ovarian cancer screening, attitudes, beliefs, UKCTOCS

## Abstract

**Background:**

Women's awareness of ovarian cancer (OC) risks, their attitudes towards and beliefs about screening, together with misunderstandings or gaps in knowledge, may influence screening uptake.

**Methods:**

In total, 21 715 post-menopausal women completed questionnaires before randomisation into the UK Collaborative Trial of Ovarian Cancer Screening.

**Results:**

In all, 42.3% correctly identified their lifetime risk of OC; 87.1% knew that a family history of OC increased risk, but only 26.7% appreciated the association with a family history of breast cancer. Although 38.2% acknowledged increased risk post-menopause, only 8.8% were aware that OC diagnoses are highest in women over 65 years. Few (13.7%) recognised the association between pregnancy and reduced OC risk or protective effects of breastfeeding (6.2%). There were common misconceptions; 37.2% thought that an abnormal cervical smear and 26.4% that oral contraception increased the likelihood of OC. Although 84.4% recognised that most ovarian masses are benign, 20.2% thought having had a benign cyst increased OC risk. Most (99.4%) believed that a high uptake of OC screening would reduce mortality and (96.2%) that screen-detected cancers would have an improved prognosis.

**Conclusions:**

The results show a need for improved public understanding about OC risks and provide important information for GPs and health educationalists about initiatives needed for future awareness, prevention and screening programmes.

In 2007, 4317 UK women died from ovarian cancer (OC), the fifth most common cancer in women (Cancer Research UK, 2009). Earlier diagnosis is associated with improved prognosis, yet many women present too late with advanced disease. The primary aim of the UK Collaborative Trial of Ovarian Cancer Screening (UKCTOCS) is the assessment of screening on mortality. The resource implications, acceptance, physical and psychosocial morbidity of screening are also being measured. In total, 202 638 post-menopausal women from the general population aged between 50 and 74 years have been recruited to the trial (Menon *et al*, 2008, 2009). Women from 13 centres in England, Wales and Northern Ireland were randomised into three groups: a no intervention group control, multimodal screening (annual screening with serum CA125 followed by transvaginal ultrasound scan as a second-line test) or annual transvaginal ultrasound. At trial entry, participants were asked about their awareness of OC risk factors and views about putative benefits of screening, to identify possible misunderstandings or gaps in knowledge that might influence willingness to adhere to screening. Such data are also valuable for health education initiatives.

Little is known about the awareness of the risks of OC or views about screening among women in the general population of the United Kingdom, although a survey commissioned in 2007 by an OC charity and Dr Foster Intelligence suggested that 59% of women were unaware of any risk factors associated with OC (Dr Foster, 2007). Most published research has been carried out in the United States (Andersen *et al*, 2002, 2004; Hensley *et al*, 2003; Salsman *et al*, 2004; Lockwood-Rayermann *et al*, 2009), which might not extrapolate to women in the United Kingdom. The methodological inadequacies of some studies have been described (Salsman *et al*, 2004). Furthermore, many reports have involved women at high familial risk of OC (Wardle, 1995; Hensley *et al*, 2003; Andersen *et al*, 2004; Tiller *et al*, 2005) who may well have different attitudes and beliefs to the general population. However, findings to date suggest that screening attendees may overestimate their OC risk (Andersen *et al*, 2002; Hensley *et al*, 2003) and that this may be associated with psychological morbidity (Robinson *et al*, 1997). Women may be unaware of links between breast cancer and OC (Andersen *et al*, 2004) and may misunderstand the purpose of screening (Brain *et al*, 2004). There may be confusion between cervical cancer and OC screening (Black *et al*, 2007; Lockwood-Rayermann *et al*, 2009) and a lack of understanding about the fallibility of screening tests (Tiller *et al*, 2005; Editorial, 2009).

In this paper, we report the knowledge and pre-randomisation beliefs of 21 715 women participating in UKCTOCS regarding OC risk, perceived risk factors, their attitudes towards screening and the associations of all these with sociodemographic, psychological characteristics and medical history.

## Materials and methods

The design of the main UKCTOCS has been described in detail (Menon *et al*, 2008, 2009). It has ethical approval from the multicentre regional and local ethics committees, and all participants signed a consent form. Between April 2001 and October 2005, 91.6% (185693/202638) women provided separate written informed consent to participate in the psychosocial arm of the trial, which examined knowledge, beliefs and attitudes to OC screening, together with psychological and sexual well being. Before recruitment and questionnaire completion, participants viewed an information DVD and participated in a group discussion on the need for a randomised trial. Baseline questionnaires were completed after recruitment, but before randomisation and returned to the psychosocial study centre by mail. Owing to a variety of methodological, economic and practical constraints, most previously reported research uses cross-sectional analysis. Measurement of the psychosocial impact of trial participation (which will be reported on trial completion) necessitates a truly prospective, longitudinal study. Therefore, two types of follow-up were initiated: first, the baseline data from a random sample across all three UKCTOCS groups (two screening and one control group) were automatically entered on a database for longitudinal follow-up, and second, the baseline questionnaires from the remaining participants were stored, but only entered onto the database for longitudinal follow-up if women were recalled during the trial for repeat tests or extra screens.

### Sample

**Figure 1:**
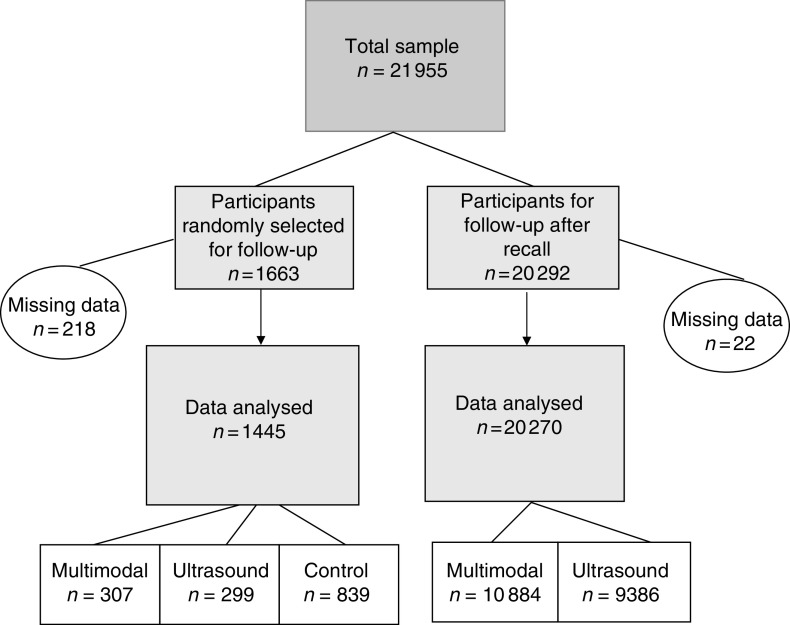
Sample description.

This paper describes data from pre-randomisation baseline questionnaires about the attitudes and beliefs of 21 715 women: 1445 randomly selected at the outset and 20 270 who were recalled for further tests (Figure 1). Thus, the majority of women described in this paper were randomised to one of the two UKCTOCS screening groups: 51.5% (11 191) to the multimodal group, 44.6% (9685) to the ultrasound group and the remaining 3.9% (839) to the control group. It should be noted that all the data about risk perception, attitudes and beliefs were provided at baseline before randomisation and any recall.

### Study measures

Participants completed sociodemographic and medical history details and four questionnaires: (1) one (see Appendix B) probing perceived OC risk, beliefs and attitudes about screening adapted from a questionnaire used previously on breast cancer screening (Fallowfield *et al*, 1990); (2) the Spielberger State/Trait Anxiety Inventory (STAI) (Spielberger *et al*, 1983), to measure anxiety proneness (trait anxiety); (3) the General Health Questionnaire 12 (GHQ-12) (Goldberg and Williams, 1988), a screening tool to determine general psychiatric morbidity or emotional distress in clinical settings or community studies and (4) Fallowfield's Sexual Activity Questionnaire (Atkins and Fallowfield, 2007). Highest education level achieved, personal and family history of breast cancer and OC and use of oral contraception were obtained from the main UKCTOCS dataset.

### Statistical analysis

Logistic regression was used for binary outcomes and proportional odds logistic regression for ordinal outcomes. The proportional odds logistic model assumes that, for any dichotomy of the ordinal scale, all odds ratios of interest, comparing the odds of observing a ‘higher’ outcome between two groups, do not depend on the cut point of the dichotomy. The multiple explanatory variables included in these models were age group, education level, centre, anxiety level, poor psychological health, partnership status, family history of OC (at least one relative: mother, daughter, sister, grandmother, granddaughter, aunt), family history of breast cancer (at least one relative: mother, daughter, sister, grandmother, granddaughter, aunt/more than one relative: mother, daughter, sister, grandmother, granddaughter, aunt), personal history of breast cancer and having used oral contraception. Education level was categorised into three groups according to the highest formal qualifications specified: tertiary education (a university degree or a nursing/teaching qualification), secondary education (O-levels, A-levels or a clerical/commercial qualification) and no formal qualifications specified. Anxiety level was categorised in three groups: low, average and high. Low and high anxiety were, respectively, defined as an STAI trait score lower or higher than one s.d. from the mean score using the sample mean (36.5) and s.d. (10.1). The GHQ score covariate was dichotomised with a ‘case’ of probable psychological morbidity defined as a GHQ score of 4 or more. The results are reported as odds ratios with 95% confidence intervals (CI). Reported results for all regression models are adjusted for centre. Analyses restricted to the random sample were undertaken, but no qualitative differences arose.

With such a large sample size, tests of the simple null hypothesis of no effect because of an explanatory variable led to many significant results. However, many of the estimated odds ratios, significant at the 5% level are close to 1 and too small to be of practical importance (Armitage *et al*, 2002, p. 91). Therefore, comments are restricted to those estimated odds ratios, which are over 1.5 or below 0.7, corresponding to an increase in odds of 50%, and the equivalent decrease on a log scale. The limits of 1.5 and 0.7 were chosen arbitrarily before examination of trial results. To avoid being overly restrictive, we have allowed ourselves some leeway in borderline cases and cases of earlier interest.

## Results

### Sociodemographic and psychological characteristics and medical history

The sociodemographic and psychological characteristics of participants, which were very similar in the random- and recall-based samples and are shown in Table 1, suggest that they were representative of the general population; 50.4% reported educational qualifications (O-levels and above), which compares with 48.4% of UK females in 2005 (aged 55–64) who have attained at least upper secondary education (OECD, 2006). The mean STAI trait anxiety score of 36.5 (s.d. 10.1) is lower than that of women attending a familial OC clinic in Scotland (mean=40.1, s.d.=9.0) (Cull *et al*, 2001), but similar to that in a UK study of women ‘at risk’ of developing breast cancer (Thirlaway *et al*, 1996). The proportion of participants (14.2%) identified as ‘cases’ (GHQ-12 score ⩾4) is similar to the 15% of ‘cases’ among females (16 years or older) in England in 2003 (Prescott and Moody, 2004).

### Perception of risk

When asked to estimate the lifetime risk of *a woman in this country* getting OC, 42.3% (95% CI: 41.7–43.0) of the 21 358 respondents gave the most accurate answer (1 in 70), whereas 50.1% underestimated the risk (1 in 500) and 7.6% overestimated the risk (1 in 12). When asked to estimate their *own* lifetime risk of developing OC, 38.9% (95% CI: 38.2–39.5) selected 1 in 100, 54.2% of 21 332 selected 1 in 500 and 6.9% selected 1 in 10. Estimation of a higher level of personal risk was associated with a personal history of breast cancer and a family history of OC and higher anxiety levels (Table 2, column 1).

Just under half of 21 379 respondents believed that they were at higher risk of developing cancers other than OC (48.3% 95% CI: 47.6–49.0), and this was considerably more likely among respondents with a personal or family history of breast cancer and also associated with a higher level of education (Table 2, column 2).

The majority (84.4% 95% CI: 83.9–84.9) of 21 552 respondents were aware that most ovarian lumps turn out to be cysts rather than cancer and 13.8% did not know. Those who answered correctly were more likely to be younger, more educated and less anxious (Table 2, column 3).

Only 8.8% (95% CI: 8.4–9.2) of 21 205 respondents were aware that the chances of an ovarian lump being cancer are highest in women aged over 65 years. Correct answers were mainly associated with having a family history of OC, older age and tertiary education (Table 2, column 4).

### Knowledge of risk factors

Participants were given a list of factors and asked to select those they thought were associated with an increased risk of developing OC (Table 3). Most participants (87.1%) were aware that a family history of OC was associated with an increased risk of developing the disease, but only 26.7% knew of the increased risk associated with a family history of breast cancer. Less than two-fifths acknowledged the increased likelihood of developing OC after the menopause and few recognised the association between pregnancy and reduced OC risk or the protective effects of breastfeeding. A fifth associated having breast cancer with increased risk. There were also misconceptions; 37.2% associated having an abnormal cervical smear with an increased likelihood of developing OC. Over a quarter of the participants mistakenly believed that taking the contraceptive pill increases the risk, and a fifth believed a benign cyst increased the risk.

Younger participants, those with a higher level of education and those with more than one relative with breast cancer had a better awareness of these risk factors (Table 4). For younger participants and those with a higher level of education, this was mainly due to their greater awareness of the risk of familial OC and the increase in risk post-menopause. Those with a higher level of education also had greater awareness of the protective effects of pregnancy and breastfeeding. The significant odds ratio for those with relatives with breast cancer was primarily due to a greater awareness of the risks associated with having family members with breast cancer and having had breast cancer.

### Views on susceptibility and screening

A quarter of 21 577 respondents (25.2% 95% CI: 24.6–25.7) had spoken to other members of their family about the risk of OC and this was more likely among those with a family history of OC than those without a family history (odds ratio: 2.76; 95% CI: 2.41–3.16). Participants were asked what they perceived to be the costs and benefits of screening and for their views on their susceptibility to OC (Table 5). The majority (99.4%) believed a high uptake of OC screening would reduce mortality and (96.2%) that screen-detected cancers would have an improved prognosis. Witnessing friends or hearing of public figures getting OC increased awareness of personal risk about developing OC for 84.8%. Only 15.2% said that coming for screening would cause them to worry unnecessarily, but over two-thirds expressed concerns about developing OC.

## Discussion

### Principal findings

Just over two-fifths of the UKCTOCS participants had an accurate awareness of lifetime population risk of OC, but half underestimated the risk. As far as personal risk was concerned, higher levels of anxiety, a personal history of breast cancer or a family history of OC were associated with a higher estimation.

With the exception of a family history of OC, there was a lack of awareness regarding specific risk factors and there were some common misconceptions. Only 26.7% knew of the increased risk of OC associated with a family history of breast cancer. Only 38.2% identified the increased likelihood of developing OC after the menopause (this despite participating in a trial where menopausal status was one of the eligibility criteria for OC screening) and <1 in 10 were aware that the chances of an ovarian mass being cancer are highest in women older than 65 years. Few recognised the association between lower parity and increased OC risk or the protective effects of breastfeeding. Even more importantly, there were mistaken beliefs; over a third associated having an abnormal cervical smear with an increased likelihood of developing OC and over a quarter of the participants believed taking the contraceptive pill increases the risk of OC.

Despite the absence of data yet showing conclusive benefit of OC screening, most participants believed that a high uptake of it would reduce mortality and that screen-detected cancers would have an improved prognosis. The recent campaigns and publicity to improve awareness about the symptoms of OC are clearly important as only 41.1% recognised that it might be possible to identify symptoms sooner than waiting for screening.

Ultrasonography is an invasive procedure and more than half (51.9%) of the participants felt that gynaecological examinations were embarrassing, which might ultimately have an effect on willingness to attend and acceptability of this form of screening. Although approximately two-thirds were concerned about getting OC, it was encouraging that few (15.2%) said that coming for OC screening would cause them to worry unnecessarily.

### Strengths and weaknesses of the study

The women participating in UKCTOCS were volunteers from the 1.2 million invited and their risk perceptions may reflect this bias. Participants' general knowledge of OC may also have been raised due to pre-recruitment information. In addition, volunteers for prevention or screening trials tend to be healthier and may be better educated and of higher socioeconomic status than the general population (Pavlik *et al*, 2000; Pinsky *et al*, 2007). Nevertheless, the formal education qualifications and GHQ scores of the UKCTOCS participants suggest that these volunteers are fairly representative of the general population and anxiety levels were not especially high. This may reflect the methods used in UKCTOCS: recruitment by random invitation using participating local authorities' registers to select participants who are more representative of the general population than those who self-refer through advertisements (Menon *et al*, 2008, 2009). Therefore, the knowledge about OC and attitudes of this large group of UK women, who were not at high familial risk of the disease, is valuable for estimating awareness and misconceptions among the general population.

When considering screening participation women need to consider their own risk of developing OC, as there are potential costs as well as benefits. The questionnaire asked about numeric estimates of lifetime risk, but the interpretation of probability information, is difficult (Woloshin and Schwartz, 1999; Lipkus, 2007) and it may be unnecessary for women to be able to quote numeric lifetime risk, as long as they understand the likelihood of developing OC compared with other diseases. Other methods may elicit different estimates.

The risk factors included in the UKCTOCS questionnaire were selected >10 years ago and other risk factors for OC have since been described including obesity, current use of hormone replacement therapy, infertility, perineal talc use and endometriosis. Recent campaigns to increase OC awareness may also have increased public understanding.

### Interpretation

The results from UKCTOCS and other international studies regarding the ability of screening to detect OC in time to provide treatment that might influence survival, the best screening method, its acceptability to patients and other costs and benefits are still awaited. If the data do support implementation, then information including the risk perceptions of individuals who agree to OC screening may be important to our understanding of factors that motivate attendance, especially as there are reported inequalities in the use of existing breast and cervical screening services (Moser *et al*, 2009). Previous research suggests that OC screening attendees may believe themselves to be at higher personal risk of and worry more about the disease than others (Wardle, 1995; Andersen *et al*, 2002). Half of the UKCTOCS participants underestimated risk. At the same time, many thought that they were susceptible: only 48% of the UKCTOCS participants believed that they were at higher risk of developing cancers other than OC, although this was a more common belief among those with a higher level of education and those with a personal or family history of breast cancer. Views about susceptibility to OC were similar to those expressed in an earlier study among women attending for breast cancer screening, as were perceptions about the costs and benefits of screening (Fallowfield *et al*, 1990). Fewer of the UKCTOCS participants than those in the breast cancer screening study said that coming for screening had made them worry about cancer. Nevertheless, approximately two-thirds were concerned about getting OC. This seems excessive, considering the actual likelihood of their developing the disease, but women's anxieties could be a reflection of their knowledge that survival is often poor.

As all the UKCTOCS participants had volunteered for the study, it is not surprising that most believed in the value of OC screening, but the majority of participants also believed that an increase in uptake would reduce mortality. This finding is perhaps surprising as women had received a detailed information sheet on the pros and cons of screening and had attended an information session during recruitment, when they were informed that the primary aim of UKCTOCS is to examine whether ovarian screening can reduce mortality. The belief that screen-detected cancers would have a better prognosis is perhaps more understandable given that this is the underlying premise on which OC screening is based. These beliefs highlight the importance of presenting balanced information about the limitations as well as the benefits of screening, which has been highlighted in recent controversies about breast screening (Welch, 2009). However, even if balanced information is provided, individuals may have selective recollection of this as well as preconceived ideas about screening initiatives.

### Implications for clinicians and policymakers

The 6-year UKCTOCS follow-up study will be able to examine whether an underestimation of population risk leads to poorer attendance for screening. As well as improving information about OC risk and screening, more research needs to be performed on ways of assisting comprehension and understanding.

If the risk of OC is underestimated in the general population, campaigns should be encouraged to increase awareness. At the same time, it is important that this should be put in perspective and women should be aware that OC is only the fifth most common cancer among women in the UK accounting for 5% of all female cancers (Cancer Research UK, 2009). Specific information to remove the confusion between cervical and ovarian screening needs to be provided and the protective effects of oral contraception should be emphasised. However, health educators do have to consider the potential confusions that can easily occur, for example oral contraception is associated with higher breast cancer risk.

Although having more educational qualifications, a family history of cancer and to some extent age were associated with improved knowledge about specific aspects of OC, there was a low level of awareness in general. Thus, the findings from this large study of UK women show the urgent need for improved public education about all aspects of OC. Opportunistic discussions with women about OC when being seen by their primary care physicians and information leaflets in appropriate settings may all contribute. These results provide important information for the development of awareness initiatives that might be needed for OC prevention and for screening programmes, if they are adopted.

## Figures and Tables

**Table 1:** Baseline characteristics of participants

**Age (years)**	***n*=21 715**
Median (range)	61 (50–74)
Mean	60.9
	
*Partnership status*	% (*n*=*21 506)*
Partner	78.4 (16 850)
No partner	21.6 (4656)
	
*Highest educational qualification*	% (*n*=*20 082)*
O-level or equivalent	7.9 (1597)
A-level or equivalent	2.3 (457)
Clerical or commercial qualification	18.4 (3691)
Nursing or teaching	7.0 (1416)
College/university degree	14.8 (2963)
None specified/or stated none of the above	49.6 (9958)
	
*STAI trait anxiety (minimum*=*20, maximum*=*80)*	% (*n*=*21 211)*
Low score (20–26)	16.7 (3547)
Average score (27–46)	66.0 (13 989)
High score (47–80)	17.3 (3675)
	
*GHQ 12 (minimum*=*0, maximum*=*12)*	% (*n*=*21 598)*
‘Case’ (score=4 or more)	14.2 (3077)
	
*Medical history*	% (*n*=*21 692)*
At least 1 relative^a^ with ovarian cancer	4.6 (999)
At least 1 relative^a^ with breast cancer	21.9 (4742)
Had breast cancer	4.0 (878)
Ever used oral contraception	58.7 (12 735)

Abbreviations: GHQ=General Health Questionnaire; STAI=Spielberger State/Trait Anxiety Inventory.

a Relatives specified: mother, daughter, sister, grandmother, granddaughter, aunt.

**Table 2:** Relationship between responses to questions regarding perception of risk^a^ and participants' characteristics

	**Odds ratio (95% CI)**			
**Covariate**	**Personal risk higher (*n*=19 082)**	**Higher risk of other cancers (*n*=19 112)**	**Ovarian lumps (*n*=19 261)**	**Cancer risk by age (*n*=18 965)**
*Age group*				
50–54	Reference			
55–59	1.00 (0.92–1.09)	0.99 (0.91–1.08)	0.95 (0.84–1.09)	1.02 (0.86–1.22)
60–64	0.95 (0.87–1.04)	1.00 (0.91–1.09)	0.79 (0.69–0.90)	1.42 (1.20–1.69)
65–69	0.92 (0.84–1.01)	0.98 (0.89–1.08)	0.67 (0.58–0.76)	2.37 (1.99–2.81)
70–74	0.84 (0.75–0.94)	0.94 (0.83–1.06)	0.47 (0.41–0.55)	2.37 (1.94–2.89)
				
*Education level*				
None specified	Reference			
Secondary	0.80 (0.75–0.86)	1.12 (1.05–1.20)	1.50 (1.36–1.65)	1.02 (0.90–1.16)
Tertiary	0.74 (0.69–0.80)	1.55 (1.44–1.68)	1.98 (1.76–2.22)	1.74 (1.54–1.96)
				
*Anxiety (STAI trait score)*				
Low	Reference			
Average	1.27 (1.17–1.38)	1.19 (1.09–1.28)	0.88 (0.78–0.99)	0.96 (0.84–1.09)
High	1.81 (1.62–2.01)	1.39 (1.25–1.55)	0.62 (0.53–0.72)	0.96 (0.79–1.17)
				
GHQ case (*vs* non-case)	1.06 (0.96–1.16)	1.12 (1.02–1.23)	0.95 (0.84–1.08)	0.84 (0.70–1.00)
Partner (*vs* no partner)	0.99 (0.93–1.06)	0.99 (0.92–1.06)	1.01 (0.91–1.11)	0.97 (0.86–1.10)
Relative with ovarian cancer	1.94 (1.70–2.21)	0.90 (0.78–1.03)	0.82 (0.69–0.98)	1.46 (1.18–1.81)
One relative with breast cancer	1.17 (1.09–1.26)	1.66 (1.54–1.79)	1.07 (0.97–1.20)	1.07 (0.94–1.21)
>1 relative with breast cancer	1.41 (1.22–1.63)	3.13 (2.65–3.70)	1.04 (0.84–1.29)	0.96 (0.73–1.25)
Had breast cancer	2.40 (2.09–2.77)	2.54 (2.16–2.99)	1.27 (1.02–1.59)	0.97 (0.75–1.25)
Ever used oral contraception	0.88 (0.83–0.94)	1.05 (0.98–1.12)	1.23 (1.13–1.35)	1.11 (0.99–1.24)

Abbreviations: CI=confidence interval; GHQ=General Health Questionnaire; STAI=Spielberger State/Trait Anxiety Inventory.

a The results correspond to a proportional odds logistic regression of the probability of estimating a higher level of personal risk and logistic regressions for the probability of answering ‘yes’ to the question ‘Do you think you are at higher risk of developing other cancers than ovarian cancer’ and the probabilities of being aware that most ovarian lumps turn out to be cysts and that the chances of an ovarian lump being cancer are highest in women aged over 65 years, respectively.

**Table 3:** Women's knowledge of risk factors^a^

	**% Responding yes (95% CI)**
(a) Has relatives with ovarian cancer	87.1 (86.7–87.6)
(b) Past menopause	38.2 (37.5–38.8)
(c) Has relatives with breast cancer	26.7 (26.1–27.3)
(d) Never pregnant	13.7 (13.3–14.2)
(e) Did not breastfeed	6.2 (5.8–6.5)
(f) Has had breast cancer	19.6 (19.1–20.1)
(g) Has had abnormal smear	37.2 (36.6–37.9)
(h) Took the pill	26.4 (25.8–27)
(i) Has had benign ovarian cyst	20.2 (19.7–20.8)

Abbreviation: CI=confidence interval.

a The questionnaire stated ‘A woman is more likely to develop ovarian cancer if she: (tick any of these you think may apply).’ The sample size is 21 377.

(a–e) are accepted risk factors, (f) is equivocal in the absence of family history and (g–i) are not risk factors.

**Table 4:** Relationship between the number of correct responses regarding risk factors^a^ and participants' characteristics

	**Odds ratio (95% CI)**
**Covariate**	**Number of correct risk factor responses**
*Age group*	
50–54	Reference
55–59	1.04 (0.97–1.13)
60–64	0.92 (0.85–1.00)
65–69	0.80 (0.74–0.87)
70–74	0.70 (0.63–0.77)
	
*Education level*	
None specified	Reference
Secondary	1.32 (1.24–1.40)
Tertiary	2.11 (1.97–2.25)
	
*Anxiety (STAI trait score)*	
Low	Reference
Average	0.97 (0.91–1.04)
High	0.82 (0.74–0.90)
	
GHQ case (*vs* non-case)	0.82 (0.76–0.89)
Partner (*vs* no partner)	1.02 (0.96–1.08)
Relative with ovarian cancer	1.15 (1.02–1.29)
One relative with breast cancer	1.28 (1.20–1.37)
>1 relative with breast cancer	1.47 (1.29–1.68)
Had breast cancer	1.04 (0.91–1.19)
Ever used oral contraception	1.08 (1.02–1.15)

Abbreviations: CI=confidence interval; GHQ=General Health Questionnaire; STAI=Spielberger State/Trait Anxiety Inventory.

a Proportional odds logistic regression of the number of correct responses regarding risk factors (listed in Table 3).

Definition of correct responses: items (a–f) endorsed, items (g–i) not endorsed. If no items were endorsed, (g–i) non-endorsements were excluded and data treated as missing.

Regression also adjusted for centre. Sample size=19 127.

**Table 5:** Attitudes to and beliefs about ovarian cancer and screening

	***N***	**Agree (strongly/a little)^a^ (%)**	**95% CI**
*Views about ovarian cancer screening*			
If more women went for ovarian screening, there would be fewer deaths from ovarian cancer	21 586	99.4	99.3–99.5
If I was found to have ovarian cancer by screening, the chances of it being cured are higher	21 590	96.2	95.9–96.4
I find gynaecological examination an embarrassment	21 526	51.9	51.3–52.6
If I look out for the symptoms of ovarian cancer, I might find something sooner than if I go for screening	21 423	41.1	40.5–41.8
If a lump is found in your ovaries, it is usually too late to do anything.	21 506	22.4	21.9–23.0
Coming for screening would/has only made me worry (unnecessarily) about ovarian cancer	21 562	15.2	14.7–15.7
			
*Views about susceptibility to ovarian cancer*			
Whenever I hear of a friend/relative or public figure getting ovarian cancer, I realise I could get it too	21 522	84.8	84.3–85.2
The older I get, the more I think about the possibility of getting ovarian cancer	21 474	53.3	52.6–53.9
There are so many things that could happen to me, it is pointless to worry about ovarian cancer	21 478	36.2	35.6–36.8
My health is too good at present even to consider thinking that I might get ovarian cancer	21 490	32.0	31.4–32.6

a Questionnaire had four choices: agree strongly, agree a little, disagree strongly, disagree a little, which were dichotomised.

## APPENDIX A

**UKCTOCS team and affiliations**

**Principle investigators:** Ian Jacobs FRCOG^1^, Usha Menon MD^1^, Stuart Campbell DSc (Lond)^2^, Lesley Fallowfield DPhil ^3^, Steven J Skates PhD^4^, Mahesh Parmar PhD^5^, Alistair McGuire PhD^6^

**Leads:** Alberto Lopes FRCOG^7^, Keith Godfrey FRCOG^7^, David Oram FRCOG^8^, Jonathan Herod MRCOG^9^, Karin Williamson FRCOG^10^, Mourad Seif PhD^11^, Ian Scott FRCOG^12^, Howard Jenkins^12^, Tim Mould FRCOG^13^, Robert Woolas MD^14^, John Murdoch FRCOG^15^, Stephen Dobbs MRCOG^16^, Nazar N Amso PhD^17^, Simon Leeson FRCOG^18^, Derek Cruickshank FRCOG^19^

**Trial steering committee:** David Luesley FRCOG^20^, Jack Cuzick PhD^21^, Julietta Patnick^22^, KK Cheng^23^, Louise Bayne^24^, Kate Law^25^, Karen Finney^26^

**Coordinating centre team:** Aleksandra Gentry-Maharaj PhD^1^, Matthew Burnell PhD^1^, Andy Ryan PhD^1^, Jatinderpal Kalsi^1^, Susan Davies^1^, Gwendolen Fletcher^1^, Sophia Apostolidou^1^, Robert Liston^1^

^1^Gynaecological Oncology, UCL EGA Institute for Women's Health, London W1T 7DN; ^2^Create Health Clinic, London W1G 6AJ; ^3^Cancer Research UK Sussex Psychosocial Oncology Group at Brighton & Sussex Medical School, University of Sussex, Falmer BN1 9PX; ^4^Department of Medicine, Harvard Medical School, Boston, MA 02115, USA; ^5^Cancer Group, Medical Research Council Clinical Trials Unit, London NW1 2DA; ^6^Department of Social Policy, London School of Economics, London WC2A 2AE; ^7^Northern Gynaecological Oncology Centre, Queen Elizabeth Hospital, Gateshead NE9 6SX; ^8^Department of Gynaecological Oncology, St Bartholomew's Hospital, London EC1A 7BE; ^9^Department of Gynaecology, Liverpool Women's Hospital, Liverpool L8 7SS; ^10^Department of Gynaecological Oncology, Nottingham City Hospital, Nottingham NG5 1PB; ^11^Academic Unit of Obstetrics and Gynaecology, St Mary's Hospital, Manchester M13 9WL; ^12^Department of Gynaecological Oncology, Derby City Hospital, Derby DE22 3NE; ^13^Department of Gynaecological Oncology, Royal Free Hospital, London NW3 2QG; ^14^Department of Gynaecological Oncology, St Mary's Hospital, Portsmouth PO3 6AD; ^15^Department of Gynaecological Oncology, St Michael's Hospital, Bristol BS2 8EG; ^16^Department of Gynaecological Oncology, Belfast City Hospital, Belfast BT9 7AB; ^17^Department of Obstetrics and Gynaecology, Wales College of Medicine, Cardiff University, Cardiff CF14 4XN; ^18^Department of Gynaecological Oncology, Llandudno Hospital, North Wales LL30 1LB; ^19^Department of Gynaecological Oncology, James Cook University Hospital, Middlesbrough TS4 3BW; ^20^Pan-Birmingham Gynaecological Cancer Centre, Birmingham B18 7QH; ^21^Head of Mathematics and Statistics (Cancer Research UK), Barts and The London, London EC1M 6BQ; ^22^Director, NHS Cancer Screening Programmes, Sheffield S10 3TH; ^23^Public Health, Epidemiology & Biostatistics, Birmingham, B15 2TT; ^24^Ovacome, London W1A,7WJ; ^25^Cancer Research UK, London WC2A 3PX; ^26^Medical Research Council, London W1B 1AL.

## APPENDIX B

**Risk perceptions questionnaire**
